# The Development of Intelligent Education and Teachers' Professional Informatization Led by an Intelligent Ubiquitous Network

**DOI:** 10.1155/2022/3997080

**Published:** 2022-10-07

**Authors:** Yuyuan Zhang, Hai Zhang

**Affiliations:** ^1^School of Information Science and Technology, Northeast Normal University, Changchun, Jilin 130117, China; ^2^School of Media Science, Northeast Normal University, Changchun, Jilin 130117, China

## Abstract

This study applies intelligent ubiquitous network technology to the field of education and leads to the benign development of intelligent education and teachers' professional informatization. This study proposes the concepts of eco-classroom, smart education, and flipped classroom and conducts practical research on the teaching effectiveness of the three elements of eco-classroom: teachers, students, and environment. Because of the problems of inaccurate positioning of data fusion, single application function, and difficulty of automatic correlation of data stored in multiple networks in the intelligent ubiquitous sensing environment, the method and path of multidimensional data fusion governance are proposed and put into practice to achieve good results in practice. Based on the characteristics of ubiquitous learning in time, space, and dimensional changes between reality and time, combined with the law of knotted learning and Bloom's mastery learning theory, the five components of the teaching model and their correlations are sorted out, and the teaching model of higher education under the vision of ubiquitous learning is constructed to achieve the goal of students' mastering knowledge and skills and promote the innovation of the education and teaching model. The model interaction site is created, a ranking system is developed, and professional standards for the new era are developed to help educators reconstruct their professional competencies. The concept and characteristics of knowledge sharing in the ubiquitous network environment are analyzed, the knowledge sharing model in the ubiquitous network learning environment is constructed, the status of knowledge sharing practice in the ubiquitous network environment is studied, and suggestions for improvement are put forward to optimize knowledge sharing in the ubiquitous network learning environment.

## 1. Introduction

The development of information technology has led to new changes in human life, and Internet+ education has brought opportunities and challenges to traditional teaching. The ubiquitous network provides the basis for ubiquitous learning, and ubiquitous learning realizes learning for “everyone, everywhere, and all the time,” that is, 4A learning, where anyone, anywhere, and at any time can learn with any tool at hand. The outbreak of the new pneumonia epidemic has posed an unprecedented challenge to traditional education. It makes up for the shortcomings of traditional methods with its flexibility, convenience, economy, and humanity and plays an important role in ensuring that teaching and learning are carried out properly [1]. How to use information technology to design teaching activities, form teaching experiences and teaching rules that can be learned from, and build a new teaching model of higher vocational education under the perspective of ubiquitous learning has strong practical significance and social value. Combined with the corresponding verification platform, the data are divided into different areas by a separate calculation method, which can reduce the error while reducing the amount of calculation.

With the development of IT, new technologies such as big data, cloud computing, the Internet of Things, and Web 2.0 are applied and interconnected, and the ubiquitous network comes into being. Under the guidance of the “learning element” theory, the ubiquitous network creates a seamless connection between IT and various terminals for educators and learners, provides personalized learning services, and realizes the sharing of resources to eventually achieve intelligent aggregation. With the support of ubiquitous network technology, ubiquitous learning has become the mainstream learning mode and is attracting widespread attention in the education technology community [2]. In the United States, Harvard University has developed a handheld device to support ubiquitous learning, aiming to test whether mobile devices can increase students' interest and improve teaching quality. In the UK, an environmental forest project was developed to integrate virtual and reality by installing mobile devices in a forest. The wisdom of information has given rise to the wisdom of education and fundamentally overturned the traditional education model [3]. Ubiquitous learning provides students with different levels of academic ability and acquisition with the resources needed for learning and the context needed for communication anytime and anywhere. The current situation of higher vocational education is not optimistic, which shows that the characteristics of higher vocational education are not fully reflected, teachers pay too much attention to the fundamentals and lack the overall consideration for students' application in the career, students cannot effectively apply the knowledge they have learned, and the traditional teaching tendency is serious, which is not conducive to the cultivation of students' comprehensive ability [4]. How to promote the construction of higher vocational education and cultivate high-quality skilled talents with the aim of employment and based on competence has become a concern for educators. Ubiquitous learning brings new opportunities for education and teaching reform in higher education institutions and achieves certain results; however, not the appearance of good learning forms can bring good learning effects, and any effective learning cannot be separated from good resource integration, teaching implementation, and teaching evaluation.

Ubiquitous learning is proposed based on ubiquitous computing technology. Ubiquitous computing is dedicated to the seamless integration between computers and the physical world to build a computing environment that is online at any time and any place. This technical feature of ubiquitous computing provides technical support for ubiquitous learning, in which learning space and physical space are integrated and learning activities are integrated with daily life. Based on the “Internet+,” emerging technologies such as micro-class, catechism, video communication, microblogging, web, jitterbug, Tencent conference, virtual reality, augmented reality, simulation, 3D, naked-eye 3D, holographic imaging, instrument remote sharing, cloud data, future classroom, and cloud lab can realize multi-screen synchronization of learning through big data analysis and Intelligent push [5]. Computers and mobile phones communicate through WiMax and TD-SCDMA, respectively, and their channels adopt a random and unified packet loss model and use Multicast to transmit data packets. The future classroom or cloud lab will have four characteristics (all-territory, all-information, all-intelligence, and fully automatic) and will become the shadow system of the classroom, etc. Vocational education is especially suitable for contextual teaching and experiential learning, while emerging technologies such as virtual reality, 3D and naked-eye 3D, and holographic imaging can largely meet the needs of contextual teaching and experiential learning, where the “immersive” virtual reality and vocational education “scenario building” theory coincide. Virtual reality scenes are a special attraction for students, who wear virtual reality glasses to reproduce spatiotemporal scenes and objects that are usually difficult to observe with the naked eye in a multidimensional display and solve the problem of high risk and high cost of training in some special training scenes.

## 2. Related Works

The concept of “ubiquitous” was first proposed by Dr. Mark Weiser, chief scientist of Palo Alto Research Center, Xerox Laboratories, California, USA, in 1991 [6]. At present, scholars' research mainly focuses on the security and privacy of information resources, related technologies, and applications. In the area of security privacy of information resources, Jurva et al. studied the cryptographic authentication scheme proposed for roaming in ubiquitous networks and proposed the implementation of triple-factor authentication to enhance security performance [7]. In the ubiquitous network environment, a “trust-level privacy protection” (PPT) mechanism is proposed to protect the privacy of entity identity by making a judgment based on the trust level of the user during the connection between the user and the service communication process. The exchange computation process is carried out to achieve data protection when users send sensitive data to the network through a gateway. Based on the research related to the ubiquitous network in-network health information quality evaluation, Long et al. established a public network health information trustworthiness evaluation index system with users as evaluation subjects, which provides a decision basis for public network health information query and selection under a ubiquitous network [8]. Bonfield et al. pointed out that users in the ubiquitous network would enhance the frequency of invisible interaction with different computing devices when the use of context-aware computing technology could make better decisions for users and provide corresponding services automatically [9].

Research results on artificial intelligence and education have been increasing in recent years. As the intelligent era continues to put forward new requirements for the talents needed by society, educational goals should also be constantly adjusted. As scholars have pointed out, the national education policy, the specific training objectives of schools, the curriculum objectives, and the determination of teaching objectives should be transformed with the advent of the intelligent era. Like this view, some scholars suggest that it is necessary to break away from the previous educational goals that focus on developing students' basic knowledge, memorization, and skilled output and instead develop learners' comprehensive abilities [10]. Some researchers believe that the presentation, carriers, and dissemination methods of educational content change with the times and that the selection, transmission, learning, and even innovation of educational content are always the key to education and teaching. Starting from the three dimensions of comprehensive human development, Chinese excellent traditional culture, and human destiny community, Zhang et al. argue that the unity of basic and developmental educational content, the docking of tradition and modernity, and the mutual transformation of internationalization and localization should be realized in the intelligent era [11]. Other scholars point out that “subjects related to the principles of AI and its applications should also be encompassed in the educational content.” Furthermore, the cross-border integration brought by AI has already broken the boundaries between disciplines, and the educational content should be reorganized due to the breaking down of barriers between disciplines. It is suggested that teachers design teaching activities from the perspective of students. Teachers and students can hold teaching seminars to exchange views and improve teaching strategies.

Research on teacher professional development has focused on three aspects: connotation, influencing factors, and development strategies [12]. In terms of the connotation of teacher professional development, some scholars regard teacher professional development as the process of teachers continuously meeting professional standards, emphasizing the group and external nature of teacher professionalism, and focusing on the process of teacher professional development, which is closer to the concept of teacher professionalization. Teacher professional development includes the enhancement of teachers' self-confidence, the improvement of their teaching skills, and the expansion and sublimation of the subject matter they teach [13]. With the rapid development of information and communication technology, the information network will also develop in the direction of a ubiquitous network, which can realize communication between people, things, and things regardless of time and place. At present, data, information, and knowledge are heterogeneous, multiple, and fragmented, so the relevance of the knowledge formed by the combination of data is also weakening. Based on the abovementioned situation, if we want to provide valuable information services to network users accurately, we need to analyze and process the many and disorganized data in the network, collect information from multiple dimensions, and then, continuously locate the valuable data that meet users' needs through multiple calibrations so that we can move forward to provide accurate and efficient information services [14]. Based on the questionnaire survey, this study will discuss the construction of an information service model in the ubiquitous network environment from the perspective of knowledge integration based on user needs.

## 3. Construction of a Model of Intelligent Education and Teacher Professional Informatization Assessment Based on Intelligent Ubiquitous Network Leadership

### 3.1. Intelligent Ubiquitous Network Model Design

In 1991, Mark Weiser, a scientist at Xerox Labs, first proposed a new model of human-computer interaction beyond desktop computing, Ubiquitous Computing, which embeds information processing into computing devices in the space around the user's life and provides information and communication services to the user collaboratively and invisibly. Based on this, Japan and Korea proposed the “Ubiquitous Network” at almost the same time, and they defined the “Ubiquitous Network” as a technological society that is armed with intelligent networks, state-of-the-art computing technologies, and other leading digital technology infrastructures [15]. The Communications Standardization Association defines a ubiquitous network as a network that provides ubiquitous information services and applications for individuals and society by realizing on-demand information services between people and things, with intelligent environment awareness and contextual processing. The ubiquitous network is a widespread network with basic characteristics of being ubiquitous, omnipresent, and omnipotent to realize that anyone and anything can connect and interact with information at any time and any place. This ubiquitous connection can perceive the real physical world more thoroughly, connect the real physical world using the network, and provide people with ubiquitous networked services. According to the fuzzy analytic hierarchy process, the initial weight value of the key quality index (KQI) of the video service in 24-time units a day obtained through expert evaluation is calculated during the evaluation period without user feedback.

(1) $$\begin{array}{c} C_{\max}=\ln\left(1\right)=0\text{.} \end{array}$$

**Figure 1:**
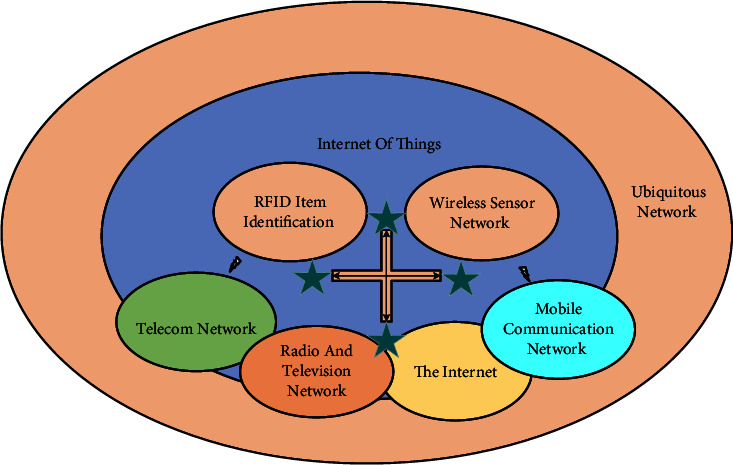
Relationship between ubiquitous networks and other networks.

The concept of the Internet of Things (IoT) was first proposed by Professor Kevin Ashton of MIT in 1999, which refers to the deployment of various information sensing devices (RFID, 2D codes, mobile communication modules, etc.) with certain sensing, computing, or execution capabilities in entities in the physical world and the transmission, collaboration, and processing of information through network facilities to achieve intelligent identification, positioning, tracking, monitoring and management of objects[16]. The infrastructure of IoT has a certain degree of overlap with the Internet. The ubiquitous network is not a new network but contains various networks such as mobile communication networks, satellite communication networks, radio and television networks, and sensor networks. It is a high degree of integration and collaboration of heterogeneous networks and covers a variety of communication technologies such as fiber optic communication, broadband access, and networking as well as sensor networks and RFID (radio frequency identification technology), Bluetooth, Wi-Fi, etc. Proximity communication technology is based on the original network, according to the needs of human social development, the corresponding network capabilities, services, and applications to increase and expand. Therefore, it can be said that a ubiquitous network is a concept that encompasses a wide range, in which sensor networks exist as a subset of IoT and IoT exists as a subset of ubiquitous networks. The relationship between ubiquitous networks and other networks is shown in Figure 1. A ubiquitous network is an evolving technology that can integrate existing network technologies, new network technologies, and other emerging technologies to enhance its performance and achieve wider coverage and higher information transmission efficiency. Learners can also communicate through collaborative learning and other methods and pass the knowledge they have mastered to other learners through oral, network, and other channels, and the learners who have received the knowledge can apply and incorporate the absorbed knowledge into their knowledge.

We first define some symbols to facilitate the formulation of the theoretical model of the proposed isolated communication scheme. In *X*_*A*_^*d*^, *X*_*A*_^*s*_*i*_^, and *X*_*A*_^*l*_*i*_^, *A* denotes either party in the communication process and has the same meaning as *B*, etc., and all denote communication entities. A and B are two networks that need to interact with data but are not directly connected, and *c*_2_, *c*_3_,…, *c*_*n*_ are independent isolated control units that together form the isolated control device *M*. The protocol between network *A* and the control unit *c*_1_ follows the communication protocol of network *A*, the protocol between the control unit *c*_*n*_ and network *B* follows the communication protocol of network *B*, and the data transfer between any two adjacent control units is based on a custom private protocol. The protocol between network *A* and the control unit *c*_*i*_ follows the communication protocol of network *A*. The protocol between the control unit and network *B* follows the communication protocol of network *B*. Data transfer between any two adjacent control units *c*_*i*+1_(*i*=1,2,…, *n* − 1) is based on a customized private protocol.

(2) $$\begin{array}{c} c_{\min}=n1n\left(1_{\max}-1_{\min}\right)\text{.} \end{array}$$

In general, the daily execution and protection of network privacy big data are composed of fixed formats and network strain structures. The traditional validation calculation method generally adds daily data information to the processing structure and uses a separated calculation method to divide the data into different areas in combination with the corresponding validation platform, which can reduce the error while reducing the calculation volume. Although this form can achieve the expected test objectives, there are still many problems in the process of practical application, which can affect the final calculation structure [17]. To address this problem, the security cycle can be combined with big data technology to build a security cycle description verification calculation model. The model can be divided into hierarchical structures based on different verification objectives, each corresponding to a different objective, and network tasks are performed one by one to delineate the scope of the cyclic security description of information and data. After the creation of the secure cyclic description integrity verification algorithm model, a bilinear verification matrix is established. The bilinear description index is calculated in the predefined network scope.

(3) $$\begin{array}{c} T=\frac{\mu +\rho }{2}-\sqrt{2}\text{,} \end{array}$$

where *T* denotes the bilinear description index, *μ* denotes the full-range validation moment value, and *ρ* denotes the ubiquitous contrast value. The derived bilinear description index is used as the validation limit criterion, the bilinear description matrix is designed, the designed structure and limitation criteria are set in the matrix, the reliability of the matrix is verified, and the actual effect of the validation calculation is further optimized by combining the setting and association of the stored range verification matrix nodes, and the design and application of the privacy big data integrity verification algorithm for ubiquitous networks is finally completed.

The QoS (quality of service) index ignores the subjective feeling of users and only reflects the evaluation of technical aspects alone, so it is difficult to reflect users' subjective experience of the services used. In this context, a new concept, quality of experience (QoE), has emerged, which indicates the user's direct feelings about the application or service used from the user's perspective. QoE completes the quantitative ranking of users' direct experience and also reflects the difference between users' expected service quality and the actual service quality obtained by users, so the optimization targeting users' QoE plays a very important role in service quality improvement.

### 3.2. Smart Education and Teacher Professional Information Assessment Model Construction

Wisdom education refers to a comprehensive, rich, diversified, and integrated education. It mainly includes education of rational wisdom, value wisdom, and practical wisdom. These three aspects are different from each other and interrelated, aiming to encourage the educated to fully explore their wisdom essence and grow into the unity of rational wisdom, value wisdom, and practical wisdom. Wisdom education in the era of “Internet+” refers to the use of modern information technology such as the Internet of Things, cloud computing, big data, and intelligence based on information to reconstruct the new order and form of education according to the innovative requirements of the information age, aiming at cultivating students' advanced thinking ability and innovation and creativity, and finally realizing wisdom teaching, wisdom learning, wisdom evaluation, and wisdom management [18]. The aim is to cultivate students' advanced thinking and innovative and creative abilities and ultimately to realize intelligent teaching, intelligent learning, intelligent evaluation, intelligent management, and intelligent services. The proposal and development of a smart classroom is the inevitable result of school education informatization focusing on teaching, classroom, and teachers' and students' activities. At present, different experts and scholars have given their views on the meaning of a smart classroom from different perspectives. Some scholars believe that from the perspective of education, a smart classroom is a process of cultivating and generating comprehensive quality with “wisdom” as the core, and its fundamental task is to “develop students' wisdom,” instead of simply “transferring knowledge.” The fundamental task is to “develop students' wisdom” rather than simply “transfer knowledge.” Some scholars believe that from the perspective of information technology, the wisdom classroom is the use of advanced information technology means to realize the information and intelligence of classroom teaching and to build a wise classroom teaching environment [11]. The proportion of use only accounts for 8%–15%, such as pictures, audio, video editing, and screen recording software. Especially as some manufacturing and art majors need virtual simulation technology as support, the technology of teachers in higher vocational colleges using these information-based teaching tools is still immature, accounting for only 2%.

**Figure 2:**
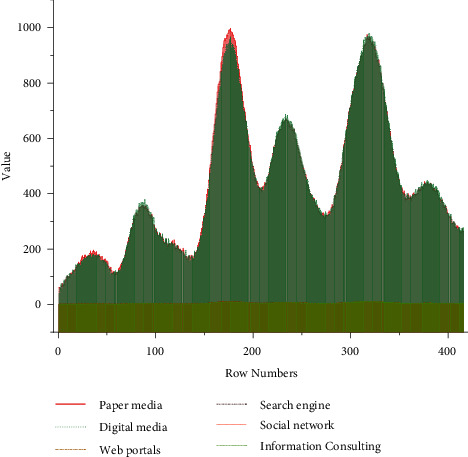
User access to information service channels.

Users' access to information services through paper-based media, digital media, portals, search engines, social networks, and information consulting are, in descending order, search engines, social networks, portals, digital media, information consulting, and paper-based media. The channels of information service access are “search engine,” “social network,” and “portal,” and the channels of information service access are “information consulting” and “paper-based media.” The most used channels are “search engine,” “social network,” and “portal,” while the less used channels are “information consultation” and “paper-based media.” This shows that people tend to use search engines such as Baidu and Bing, social networks such as Weibo and WeChat, and portals such as Today's Headlines and Sohu to obtain information services; paperless information services and online information services occupy a large market in the current information services. The results of users' access to information service channels are shown in Figure 2.

The survey data on the type of information services users obtain shows that nearly 85% of users obtain information services in the knowledge category, followed by news, facts, information, and data, and the percentage of users in these three categories is between 60% and 70%, which is the same. The survey data on the scope of information services obtained by users show that 80.1% of users obtain information services for learning, 76.98% for work, and less for entertainment and life, 65.95% and 68.35%, respectively. From the survey results, users obtain information services of various aspects and different types more frequently, among which the highest is knowledge-based services, which indicates that the types of information services required by users are diversified, and most users' needs for information services are focused on knowledge acquisition in study and work. It overcomes the limitations of the traditional teaching mode in space and time and makes up for the shortcomings of the traditional mode with its advantages of flexibility, convenience, economy, and humanization and plays an important role in ensuring normal teaching work.

**Figure 3:**
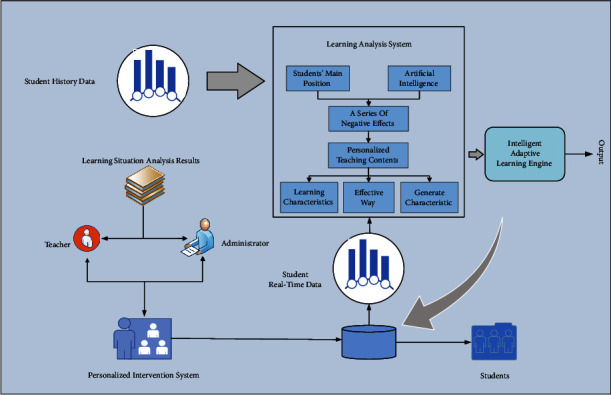
Structure diagram of the intelligent teaching system.

In the process of students learning new knowledge, the teacher is still the main body of the teaching process. Therefore, except for new teaching modes such as the “smart classroom,” most traditional classrooms still adopt the indoctrination teaching method of “teacher speaks and students listen.” In most cases, students become passive learners, which leads to a lack of clear goals, motivation, and poor independent learning ability, which hinders the development of personalized teaching [19]. At the same time, in such a teaching process, the knowledge imparted to students by teachers is mainly based on basic theories and methods and strategies, ignoring students' autonomy and motivation. Overall, the situation in which the students' main position is not prominent is becoming more serious, and this brings a series of negative effects. The application of artificial intelligence in teaching has changed this problem. For example, artificial intelligence can provide students with personalized teaching content according to their learning situation and generate characteristic models with different learning characteristics to match learners under the arrangement of big data, which provides an effective way to realize personalized teaching. The intelligent teaching system is an important foundation for personalized teaching. The structure of the intelligent teaching system is shown in Figure 3.

To understand the feasibility of user participation in the ubiquitous network information organization mechanism, the project team investigated and analyzed the motivation and influencing factors of user labeling behavior and user feedback behavior, respectively, through questionnaire research. In building the ubiquitous web information organization mechanism, users should be guided to participate in information labeling. In addition, the tags used by each user should be collected and analyzed, and users' preferences should be classified to users with similar preferences through clustering so that when users label certain information, the tags used by other users in the same group with the same information will be recommended to the user. When a user annotates a piece of information, the high-frequency tags used by other users in the same group will be recommended to the user. To improve the accuracy of annotation, when a user annotates a piece of information, a list of standardized words can also be used to recommend the standardized words of the tags annotated by the user to facilitate the user's selection. It provides personalized learning services, realizes resource sharing, and finally, achieves wisdom aggregation.

(4) $$\begin{array}{c} \varpi_{t}^{i}\left(k\right)=\varpi_{t,i}\left(k\right)-\varpi_{t,i}\left(l\right)\text{.} \end{array}$$

**Figure 4:**
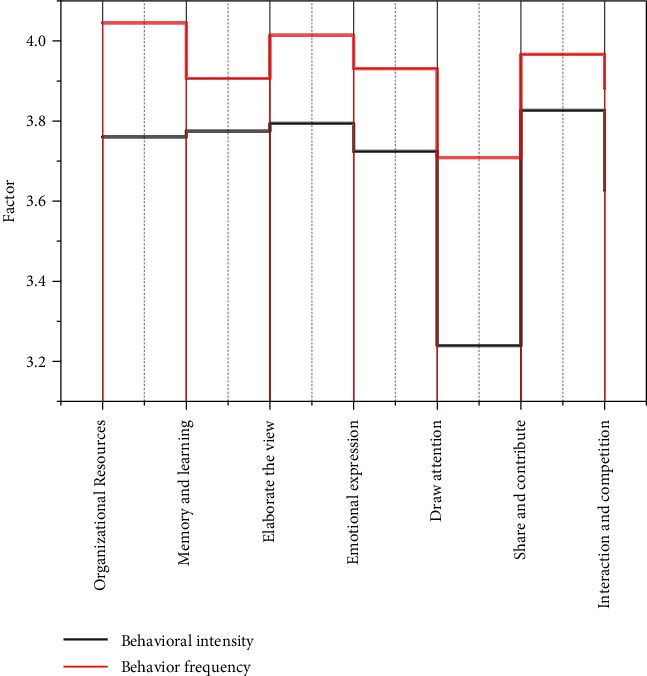
Relationship between motivation and behavioral intensity and frequency of the behavior.

The communication between learners and learners is more extensive and rapid in a network learning environment. Learners are both the recipients and transmitters of knowledge, and each learner has a different level of knowledge, so learners can communicate with each other through collaborative learning and pass their knowledge to other learners through oral and network channels. The learners who have received the knowledge apply and incorporate the absorbed knowledge into their knowledge, i.e., realize the internalization of knowledge, and then pass it to other learners in a certain form of circulation [20]. In this process, if the knowledge exchange between learners applies to the learning platform of some organizations, it will realize the enrichment and perfection of the knowledge base of this learning platform at the same time, and more learners can learn, as shown in Figure 4. In the website that attracts users to organize information, more time-based tags and long tags should be set in the recommended tags. User-labeled consultation or help function should be set on the website. The survey shows that the intensity and frequency of users' behaviors vary depending on their motivation for tagging, and users motivated by “organizing information resources” have higher intensity and frequency of behaviors. Therefore, to enhance users' motivation to mark up information, the website should try to improve users' information literacy or help users with low information literacy to master the method of marking up.

After receiving the prestudy materials from the teacher, students can complete the prestudy tasks within the specified time. Teachers can monitor students' real-time progress through the relevant platform, quickly understand and analyze learning data, help students understand the problems and error-prone knowledge that occurred during the prestudy, and create a learning record. Students can express their ideas, discuss problems encountered during the prestudy process with the teacher, ask questions or make comments, and suggest teaching activities that the teacher can design from the student's perspective. Teachers and students can hold teaching workshops to interact with each other's perspectives and improve teaching strategies. Teachers can also communicate with each other to modify and adjust their lesson plans based on the problems students encounter in their learning. Once students have completed their prestudy, they can provide feedback on their learning behaviors on the learning platform as part of the online learning assessment before class. Feedback allows students to assess themselves more objectively, check for gaps, and grasp learning priorities. Ubiquitous learning provides students with different learning abilities and acquisition levels with the resources and communication needed for learning anytime, anywhere.

## 4. Analysis of Results

### 4.1. Intelligent Ubiquitous Network Model Performance Testing

**Figure 5:**
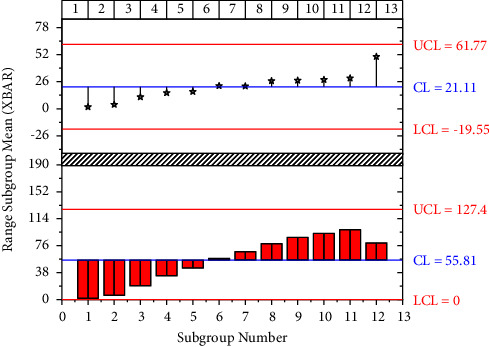
Model performance test results.

According to the actual verification requirements, the initial extension range is set and the value of the expert private key is added as the restriction target of network big data in the range and the IDB-RDIC security overlap extension verification standard is set. The final setting of the IDB-RDIC security overlap extension verification criteria can be completed. At the same time, the IDB-RDIC security overlap extension verification procedure is combined with the ubiquitous network and the corresponding protocol instructions are added. The traditional verification calculation method generally adds daily data information to the processing structure and uses a separate calculation method to divide the data into different areas in combination with the corresponding verification platform, which reduces the amount of calculation while reducing errors [21]. Although this form can achieve the expected test objectives, there are still many problems in the process of practical application, which can affect the final calculation structure. To address this problem, the security loop description verification calculation model can be constructed by combining big data technology. To ensure the reliability of the integrity verification computation, this test associates a trusted third party on the test server. Different values are added for different complexities and storage complexities, and the changes in the network are captured in combination with DPDP and DRDA verification platforms. The packets used for the test were added to the test model and tested in different time frames, resulting in the following results shown in Figure 5.

Learning activities are integrated into daily life, and elements such as learning objects, learning activities, and real-world problems in physical space can be linked together through contextualized experiences, and learning effects can be improved through ubiquitous technology. In this study, we take single-user multiterminal as the application scenario and video service as an example and further illustrate the process of QoE calculation using this improved FAHP evaluation method through Matlab simulation. User A has three terminals, TV, laptop, and cell phone, with TV as the master terminal and computer and cell phone as the slave terminals. Currently, user A watches HD TV through TV, but the video transmission rate of a single link is too low, so he improves the viewing effect by transmitting video streams through a computer and cell phone together. The TV is connected to the network through Wi-Fi, and the set bandwidth is 3 Mbps with 10 ms delay; the computer and cell phone communicate through WiMax and TD-SCDMA respectively, and their channels adopt the random uniform packet loss model and Multicast method to transmit data packets. In the simulation environment, the transmission rate is set to 1.8 Mbps and 4 Mbps, respectively, and the time delay is 10 ms. The BER is set to 10^−4^ for all three networks.

By expert evaluation, FAHP is used to calculate the initial weights of each KQI of objective factors in the 1st evaluation cycle, and *w*_*t*,*i*_(1) denotes the initial weight of the *i*-th KQI of the *t*-th evaluation time unit in the 1st evaluation cycle, (1) denotes the 1st evaluation cycle, *t* denotes the *t*-th evaluation time unit, and the value range is [1, 12]; the subscript *i* denotes the *i*-th KQI, and the range is [1, *M*]; *M* is the number of KQI; and *t* and *i* are integers greater than 0. In terms of subjective factors, user feedback is classified according to the content of user feedback, and the proportion of each type of user feedback to the total feedback in each evaluation time unit in the evaluation cycle is calculated, i.e., the feedback rate of each type of user feedback, which is expressed by *λ*_*t*,*i*_(*k*) as the feedback rate of the *i*-th type of user feedback in the *t*-th time unit of the *k* evaluation cycle.

(5) $$\begin{array}{c} \lambda_{t,i}\left(k\right)=B_{t,i}\left(k\right)\times \frac{b_{t,i}\left(k\right)}{2}\text{,} \end{array}$$

where *b*_*t*,*i*_(*k*) is the *i*-th type of user feedback in the *t*-th evaluation time cell of the *k*-th evaluation cycle; *B*_*t*,*i*_(*k*) is the total user feedback in the *t*-th evaluation time cell of the *k*-th evaluation cycle; *k* represents the *k*-th evaluation cycle and takes values in the range [1, *L*]; the subscript *i* represents the *i*-th type of user feedback and takes values in the range [1, *i*]; *L* is the number of evaluation cycles; and *i* is the number of types of user feedback.

**Figure 6:**
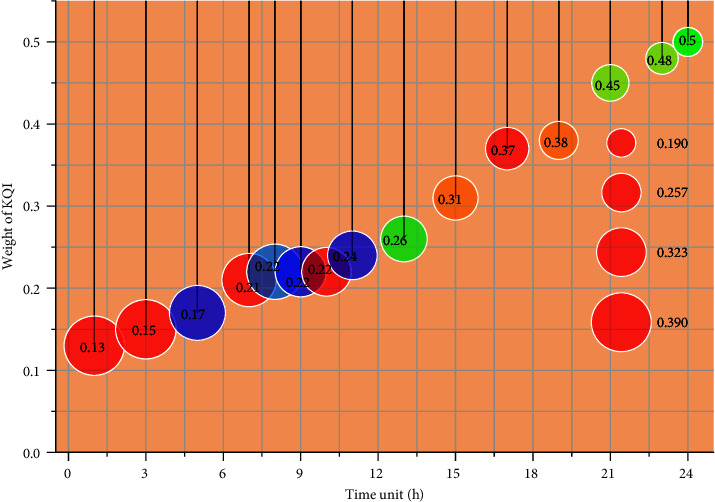
Evaluation cycle KQI initial weights.

The FAHP method is used to calculate the initial weight of each KQI: *ϖ*_*t*,1_(1), *ϖ*_*t*,2_(1), *ϖ*_*t*,3_(1) . In this calculation process, the fuzzy judgment matrix of each KQI is obtained by a two-by-two importance comparison according to the overall objective, and the weight value of each KQI is finally calculated. Taking the evaluation time unit of the evaluation cycle as an example, the fuzzy judgment moments are constructed by comparing the relative importance of each KQI among each other according to the expert evaluation first [22]. The combined importance of each KQI is calculated separately, and the normalized weight results of each KQI are finally calculated. According to the calculation steps of the fuzzy hierarchical analysis method, the initial weight values of each KQI in this evaluation time unit can be obtained. According to the fuzzy hierarchical analysis method, the initial weights of KQI of video service key quality indexes obtained by expert evaluation for 24-time units of a day in the evaluation cycle without user feedback are calculated, and the initial weights of KQI of video service key quality indexes in the first evaluation cycle are shown in Figure 6. It is a high degree of integration and collaboration of heterogeneous networks and covers a variety of communication technologies such as optical fiber communication, broadband access, and networking as well as sensor networks and short-range communication technologies such as RFID (radio frequency identification technology), Bluetooth, and Wi-Fi.

As can be seen in Figure 6, within the 7th evaluation time unit, users watch the video, and the performance of the network is relatively satisfactory from requesting playback to successful playback. The key quality indicator that users are most concerned about at this time is the video service access success, followed by the video service establishment time and, finally, the service interruption rate. According to this simulation result, the relative importance of several factors affecting the QoE of user experience can be seen. Therefore, in the virtual reconfiguration system, within a certain evaluation unit, we can focus on optimizing the key quality index with a higher weight value as an optimization target of interterminal cooperative reconfiguration so that the system can be optimized in a targeted manner to ensure that the index value is kept within a more reasonable performance range and, finally, to better improve the quality of user experience.

### 4.2. Simulation Test of the Smart Education and Teacher Professional Informatization Assessment Model

**Figure 7:**
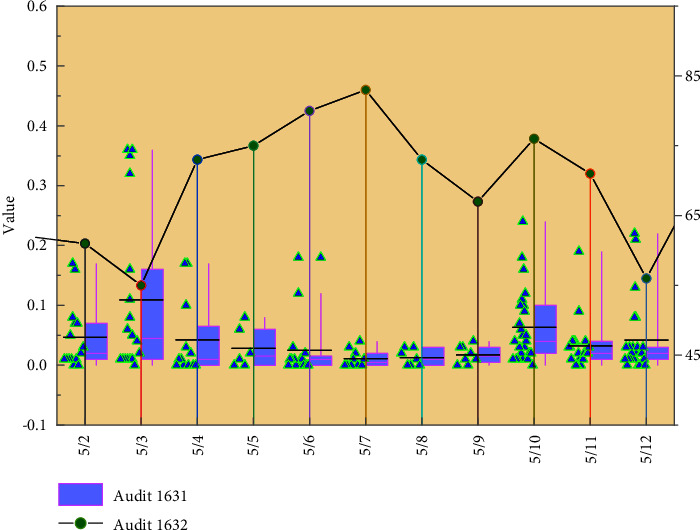
Persistence of interest in classes in the control and experimental classes.

To ensure that the experimental data were not accidental, the experiment adopted a “double-test and double-group” analysis, using SPSS data processing software to process students' performance, and a combination of qualitative and quantitative methods to evaluate students' academic performance. The experimental class was evaluated based on platform experience, online tests, advanced homework completion, and classroom performance, and the overall evaluation was combined with the project performance results, while the control class was evaluated based on traditional examination results. The questionnaire survey showed that 67% of the students accepted and agreed with the flipped classroom teaching model in the pan-learning environment and thought that their interest in learning had increased, while 31.5% of the students thought the effect was average or even smaller. As shown in Figure 7, students in the experimental class had a longer duration of interest in learning in class, indicating that the flipped classroom model in the ubiquitous learning environment was more effective in making students concentrate and increase their learning autonomy, thus effectively improving their academic performance. Combined with the corresponding verification platform, the data are divided into different areas by a separate calculation method, which can reduce the error while reducing the amount of calculation.

**Figure 8:**
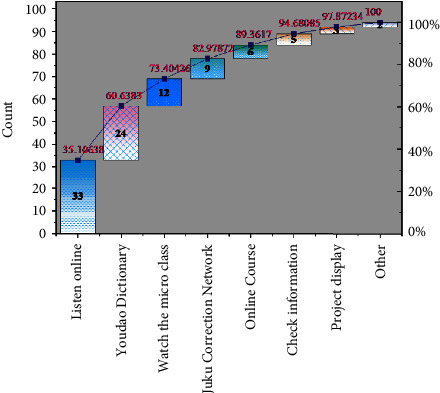
Independent learning in a ubiquitous learning environment.

Under the arrangement of big data, a feature model of different learning features that matches the learner is generated, which provides an effective way for the realization of personalized teaching. The research team investigated the independent learning situation of students in the experimental class under the ubiquitous learning environment. As shown in Figure 8, students have certain information literacy and use the Internet to complete teacher-assigned exercises, as shown by the highest frequency of using online listening, microlearning, and Youdao Dictionary to check words, while the ability to use rich Internet resources for screening is poor, as shown by the low frequency of using online courses. This indicates that students' awareness of independent learning has increased significantly in the ubiquitous learning environment, but they still need teachers' effective guidance in filtering information resources.

The subject group conducted a questionnaire on the application of information technology in teachers' teaching. The questionnaire showed that 86.2% of the teachers were able to use information technology teaching tools proficiently, including basic office software and resource search engines; 62.5% of the teachers were able to use diversified intelligent teaching evaluation tools such as process evaluation scales and student electronic file bags to monitor the teaching process. However, only 8%–15% of teachers use some specialized information-based teaching software, such as pictures, audio, video editing, and video recording software. Some manufacturing and art majors need virtual simulation technology as support, and teachers in higher education institutions are not mature enough to use these information-based teaching tools, accounting for only 2%. This shows that teachers' information literacy is limited to simple information processing in the ubiquitous learning environment and there is still room for further improvement in optimizing information resources for recreation. In the questionnaire, 87.5% of the teachers were willing to receive training on information-based teaching. Nearly 85% of the users who obtain information services belong to the knowledge category, followed by news, facts and data, and data. The percentage of users in these three categories is between 60% and 70%, which is the same.

Classroom teaching for smart education is the use of emerging technologies such as mobile technology, smart technology, and data analysis (mining) technology to computerize classroom teaching so that learners can learn efficiently and conveniently. As information technology floods classroom teaching and learning, it is especially important to ensure the ultimate effectiveness of learning. In the smart classroom, first, students should be encouraged to actively think and answer the questions set by the teacher in the classroom so that learners can master the main contents of the lesson within a limited time; second, the questions must be designed with new ideas and objectives so that students can better understand and reinforce the learning content of the lesson by answering the questions; through real-time questions and feedback, learners can combine awareness, understanding, memory, learning, and creativity. Finally, the instructional design must be clear and focused so that students can understand the key points and difficulties of the lesson briefly. In the information age, learners should always keep a fresh eye, which not only meets the needs of students' fragmented learning in the information society but also is the inevitable trend of classroom teaching reform.

## 5. Conclusion

This study constructs an assessment model of smart education and teacher professional informatization with the help of an intelligent ubiquitous network. The ubiquitous network, a product of the times, is used to lead the benign development of smart education and teacher professional informatization. Knowledge fusion technology is used to construct a ubiquitous network information service model that meets users' needs, and specific construction strategies are proposed to provide some theoretical references for later research and optimization of information service models in the ubiquitous network. The hash ratio of the privacy big data integrity verification algorithm designed in this study is relatively high, which indicates that such an algorithm has a better practical application effect and less error and has high application value. Although ubiquitous learning has overcome the limitation of time and space to a certain extent and has many advantages such as applying anytime, anywhere and being convenient, simple, effective, vivid, and interesting, there are still many shortcomings. In the ubiquitous learning environment, knowledge and learning methods are more fragmented than in traditional teaching, learning is easily disturbed, and the acquired knowledge is scattered, thus affecting the learning effectiveness, which makes the ubiquitous learning mode inevitably have certain limitations.

## Data Availability

The data used to support the findings of this study are available from the corresponding author upon request.
